# 576 Hypermetabolism Exacerbation with Increased White Blood Cell Counts in Patients with Severe Burns

**DOI:** 10.1093/jbcr/iraf019.205

**Published:** 2025-04-01

**Authors:** Beth Shields, Leopoldo Cancio

**Affiliations:** United States Army Institute of Surgical Research Burn Center; United States Army Institute of Surgical Research Burn Center

## Abstract

**Introduction:**

Severe weight loss after large burns has been previously described when the hypermetabolic response is not met with adequate nutritional provisions. We have been able to minimize this weight loss in most patients but have identified unexpected weight loss in some. The goal of this analysis was to identify predictors of this weight loss.

**Methods:**

This retrospective descriptive study was approved by the local institutional review board and included adult patients with at least 20% TBSA burns admitted between September 2017 and February 2023. The Milner equation was primarily used to estimate caloric goals, and nitrogen balance studies were used to determine protein goals. Oxandrolone and propranolol were routinely given to promote anabolism and lower the resting energy expenditure, respectively. In this analysis, patients with limb amputations, extensive fascial excision, neuroleptic malignant syndrome, malabsorption, and those who did not have a dry weight on admission (and for whom a pre-injury dry weight could not be obtained) were excluded. Energy intake from all sources was recorded from admission to the time at which the lowest near-dry weight was achieved, around the time of wound healing (defined as < 10% TBSA open) when wound healing was achieved. Edematous weights were adjusted as appropriate using the equation published by this group. Data were evaluated using JMP® (Version 13.0.0, SAS Institute, Inc. Cary, NC). Variables on univariate analysis with p< 0.1 were then entered into multivariate analysis; p< 0.05 was considered significant in the final model.

**Results:**

There were 72 patients who met the criteria for this study: 39 ± 16 %TBSA, 43 ± 15 years old, 22% female. A median weight loss of 3 kg (IQR: 1-7) occurred over 29 days (IQR: 20-50). Most (79%) patients experienced < 10% weight loss, whereas 4% sustained >20% weight loss. Univariate analysis for association between weight loss and age, sex, burn size, peak white blood cell count (WBC) in 103/uL, and caloric deficit was performed. Factors with p< 0.1 included WBC and caloric deficit, both of which remained significant in the multivariate analysis (p < 0.01). The resulting equation is: weight loss (in kg) = -3.1549 + 0.2397 x WBC in 103/uL + 0.00008 x caloric deficit (R2=0.52).

**Conclusions:**

We identified WBC and caloric deficit as correlates of weight loss. This indicates a further increase in metabolism above the expected hypermetabolic response when there is an elevation in WBC, likely reflecting concurrent sepsis or other processes driving systemic inflammation. Further research should focus on frequent metabolic cart study measurements in patients with elevated WBC counts to further elucidate this relationship.

**Applicability of Research to Practice:**

The equation determined by this study can be used to aid in minimizing weight loss by increasing caloric intake based on peak WBC

**Funding for the Study:**

N/A

